# Undiagnosed Maternal Myotonic Dystrophy Type 1 Revealed by Congenital Myotonic Dystrophy in the Neonate

**DOI:** 10.7759/cureus.105918

**Published:** 2026-03-26

**Authors:** Riku Suzui, Ikumi Wada, Motonori Matsubara, Dai Kataoka

**Affiliations:** 1 Obstetrics and Gynecology, Toyooka Hospital, Toyooka, JPN; 2 Neonatology, Toyooka Hospital, Toyooka, JPN

**Keywords:** congenital myotonic dystrophy, hypogammaglobulinemia, maternal neuromuscular disorder, myotonic dystrophy type 1, polyhydramnios, prenatal diagnosis

## Abstract

Polyhydramnios and fetal morphological anomalies, such as micrognathia and clubfoot, are findings that often suggest fetal hereditary syndromes. However, these findings may also be associated with undiagnosed neuromuscular disorders in the mother, particularly myotonic dystrophy type 1 (DM1). Maternal hypogammaglobulinemia and neonatal findings in prior pregnancies may represent overlooked diagnostic clues. We report a case in which a postnatal diagnosis of congenital myotonic dystrophy (CDM1) in the neonate led to suspicion of undiagnosed maternal DM1, with retrospective recognition of suggestive findings in the first child. A gravida 2, para 1 pregnant woman in her 30s with a history of cesarean section was under follow-up at our hospital. Her first child had exhibited mild hypotonia, low Apgar scores, slow feeding, and hypogammaglobulinemia at birth. The mother was also found to have hypogammaglobulinemia, but no definitive diagnosis was reached at that time. In the current pregnancy, polyhydramnios (amniotic fluid index (AFI) 27.8 cm) was identified at 33 weeks of gestation, and fetal ultrasonography revealed micrognathia and clubfoot. Severe polyhydramnios (AFI 40 cm) with frequent uterine contractions prompted cesarean section at 36 weeks and 5 days. The infant (birth weight 2601 g; Apgar scores 1/4) had no spontaneous breathing and exhibited marked hypotonia, requiring immediate intubation and NICU admission. Investigation prompted by the infant’s severe hypotonia revealed grip myotonia in the mother during a detailed maternal interview. Genetic testing of the infant identified approximately 1,500 CTG repeats in the *DMPK* gene, confirming CDM1. Maternal genetic testing has not yet been performed. When polyhydramnios is accompanied by fetal anomalies, such as micrognathia and clubfoot, the possibility of an undiagnosed maternal neuromuscular disorder should be considered. Neonatal hypogammaglobulinemia may reflect maternal hypogammaglobulinemia associated with DM1 rather than an intrinsic neonatal immune defect, and subtle findings in prior pregnancies may represent missed diagnostic opportunities. A thorough maternal history, combined with attention to nonspecific maternal clinical features, may contribute to earlier diagnosis, more accurate prenatal counseling, and improved perinatal outcomes.

## Introduction

Polyhydramnios is a common obstetric finding associated with a variety of fetal and maternal conditions. Fetal etiologies include structural anomalies, chromosomal abnormalities, and hereditary syndromes [1]. Among these, fetal craniofacial anomalies, such as micrognathia, and musculoskeletal anomalies, including clubfoot, frequently raise suspicion for hereditary syndromes or chromosomal abnormalities [2,3]. Consequently, prenatal evaluation is typically focused on the fetus, with the primary aim of identifying fetal structural or genetic abnormalities.

However, the underlying etiology may be related not only to fetal conditions but also to maternal neuromuscular disorders. Some of these may remain undiagnosed until pregnancy or the perinatal period. Myotonic dystrophy type 1 (DM1) is an autosomal dominant neuromuscular disorder caused by CTG repeat expansion in the DMPK gene. Congenital myotonic dystrophy (CDM1) is the most severe form, and there have been reports of transmission from mothers with mild symptoms or who were unaware of their condition [4].

Because maternal symptoms may be subtle, it is difficult to suspect CDM1 during pregnancy unless careful attention is paid to the maternal history and physical findings. Delayed recognition of the maternal condition may lead to diagnostic delay, inaccurate prenatal counseling, and difficulty in neonatal resuscitation due to delivery under inadequate preparedness. Furthermore, nonspecific clinical findings, such as hypogammaglobulinemia in the mother, may also serve as important diagnostic clues [5]. We report a case in which prenatal polyhydramnios, fetal micrognathia, and clubfoot led to a postnatal diagnosis of CDM1 in the infant, consequently raising suspicion of undiagnosed DM1 in the mother. This case highlights the importance of taking a thorough maternal history when fetal anomalies and polyhydramnios are present.

## Case presentation

The patient was a gravida 2, para 1 pregnant woman in her 30s. In the previous pregnancy, an emergency cesarean section was performed for failure to progress. The first child was a female infant, with Apgar scores of 3 at 1 minute and 7 at 5 minutes, and an umbilical artery blood pH of 7.269. The infant did not cry immediately after birth; crying was elicited with bag-mask ventilation and stimulation. Mild hypotonia was noted, and the infant was admitted to the NICU for management of neonatal asphyxia. After birth, supplemental oxygen was required in an incubator, but oxygen was discontinued on day of life 1. Feeding was slow from the beginning of hospitalization, but oral intake gradually improved, and the infant was discharged on day of life 15 after confirming adequate feeding. Blood tests performed immediately after birth revealed hypogammaglobulinemia in the first child (immunoglobulin G (IgG) 482 mg/dL). A repeat test on day of life 4 also showed a low IgG level of 468 mg/dL. Workup of the infant did not reveal a specific cause, and an undiagnosed primary immunodeficiency in the mother was suspected. Maternal blood tests were performed, revealing hypogammaglobulinemia in the mother as well (IgG 429 mg/dL; reference range: 870-1700 mg/dL). An internal medicine evaluation was conducted, but it did not reach a definitive diagnosis. At three months postpartum, the mother’s IgG had improved to 661 mg/dL, showing a gradual improvement trend, and follow-up observation was discontinued.

**Figure 1:**
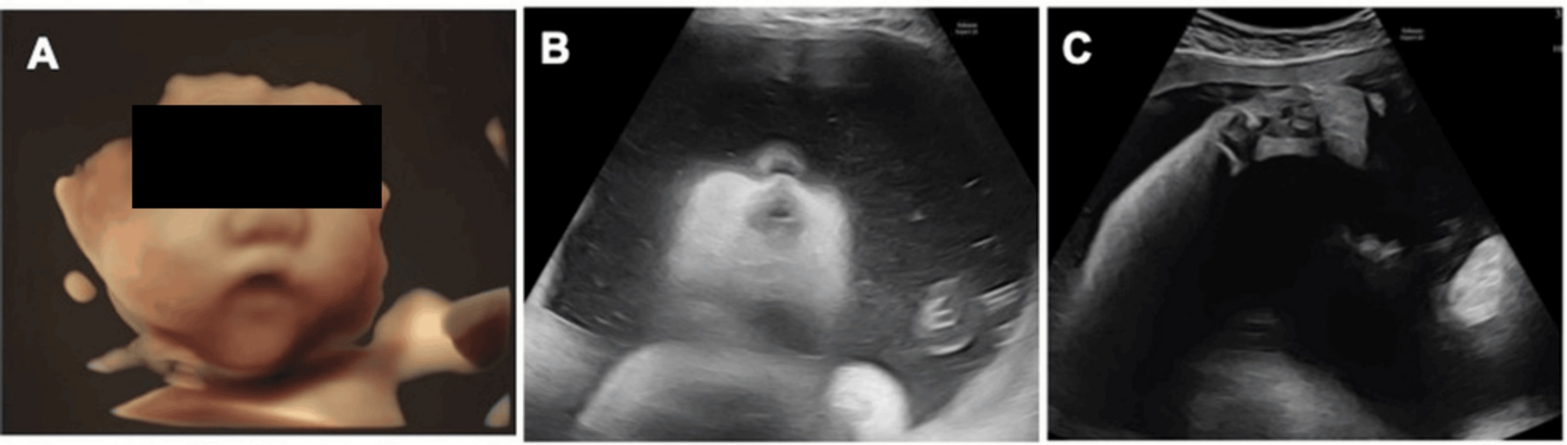
Fetal ultrasonographic findings at 33 weeks of gestation (A, B) Micrognathia; (C) Clubfoot

In the current pregnancy, the patient conceived naturally with her second child and was followed at our hospital. The pregnancy was uneventful until the third trimester. At 33 weeks of gestation, ultrasonography revealed polyhydramnios (amniotic fluid index (AFI) 27.8 cm), and detailed fetal ultrasonography demonstrated micrognathia and bilateral clubfoot. These prenatal ultrasonographic findings are shown in Figure 1.

Fetal movements and a gastric bubble were confirmed, and no other structural anomalies were detected. Based on these findings, the patient and family were counseled about the possibility of a hereditary syndrome as a differential diagnosis, and a prenatal visit by a neonatologist was conducted. During follow-up, the amniotic fluid volume progressively increased. At 36 weeks and 2 days of gestation, severe polyhydramnios (AFI 40 cm) with frequent uterine contractions was noted, and the patient was admitted for management. Given the history of a previous cesarean section and the onset of painful uterine contractions, the risk of uterine rupture was considered, and cesarean section was performed at 36 weeks and 5 days of gestation. A male infant weighing 2601 g was delivered. Apgar scores were 1 at 1 minute and 4 at 5 minutes, with an umbilical artery blood pH of 7.206. The infant had no spontaneous respirations and exhibited marked generalized hypotonia at birth. Immediate neonatal resuscitation, including endotracheal intubation, was performed, and the infant was admitted to the NICU.

**Figure 2:**
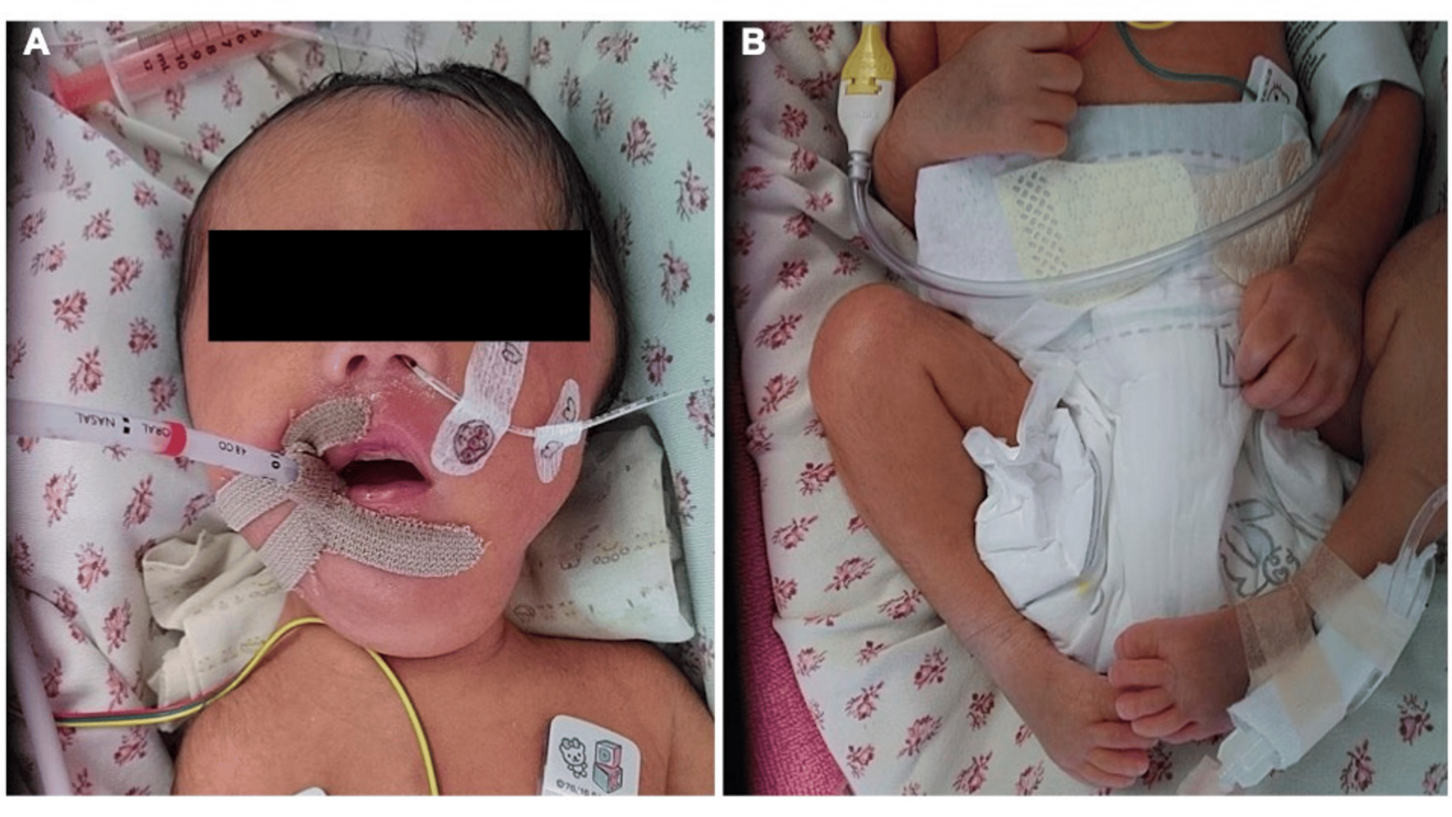
Postnatal neonatal findings (A) Neonatal facies (B) Bilateral clubfoot

After birth, the infant was found to have micrognathia and bilateral clubfoot in addition to severe hypotonia. The postnatal findings are shown in Figure 2.

Hypogammaglobulinemia (IgG 350 mg/dL) was noted. A neuromuscular disorder was suspected based on the hypotonia. During this process, a detailed maternal interview was conducted again, which revealed that the mother had experienced difficulty releasing her grip after clenching her hand (grip myotonia). Grip myotonia was subsequently confirmed on physical examination. The mother had not previously recognized this symptom as abnormal. Based on these findings, congenital myotonic dystrophy (CDM1) was suspected, and genetic counseling was provided to the mother and family. Subsequent genetic testing of the infant identified approximately 1,500 CTG repeat expansions in the DMPK gene, confirming the diagnosis of CDM1. The family was counseled regarding genetic testing for the mother and first child, as well as future pregnancies. Genetic testing has been recommended; however, the family has not yet decided to proceed.

**Figure 3:**
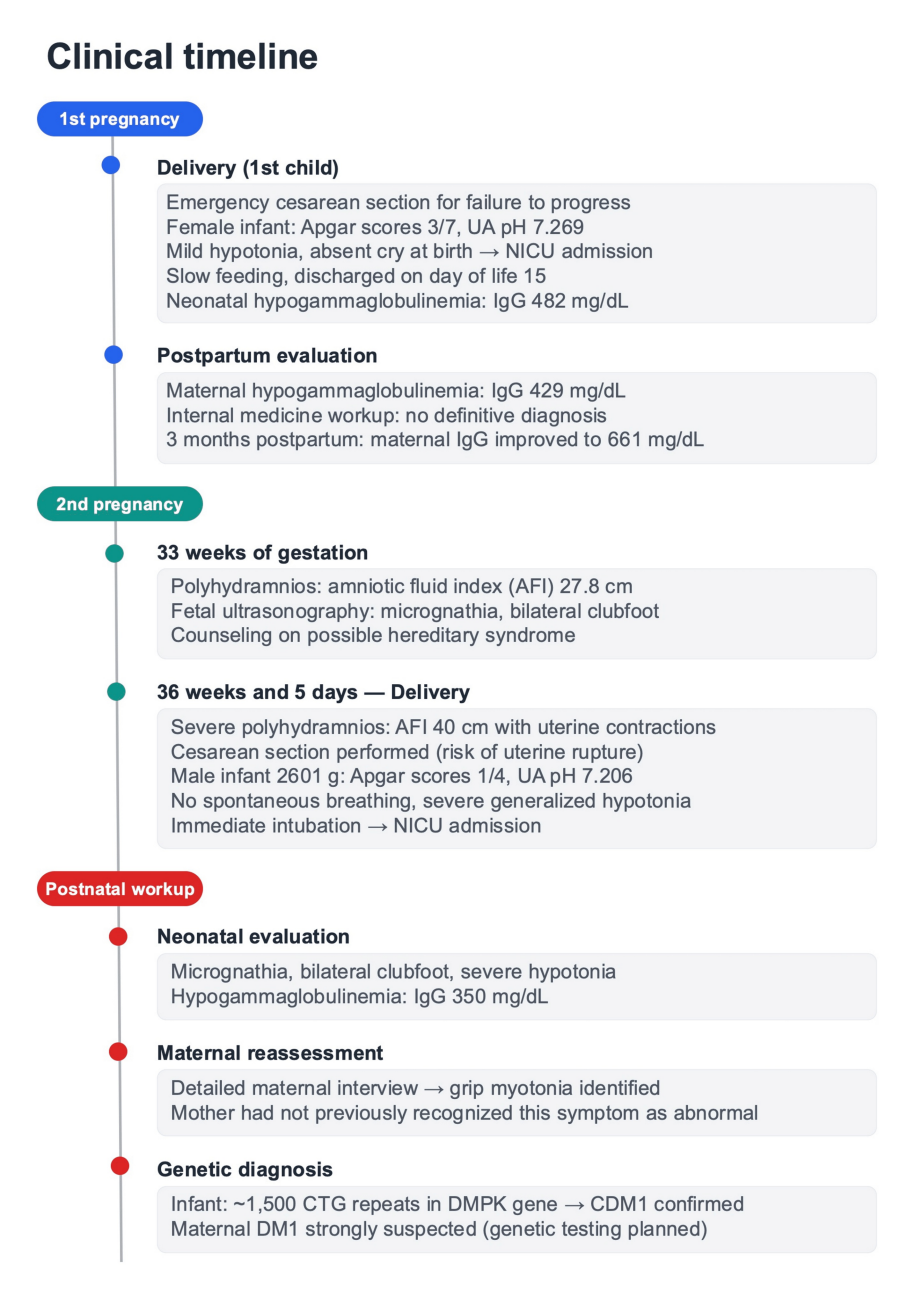
Clinical timeline summarizing both pregnancies, neonatal findings, and maternal evaluations The timeline illustrates the clinical course across the first pregnancy, second pregnancy, and postnatal workup, highlighting key findings that were retrospectively recognized as potential diagnostic clues for maternal DM1.

A clinical timeline summarizing both pregnancies, neonatal findings, and maternal evaluations is presented in Figure 3.

## Discussion

In the present case, polyhydramnios accompanied by fetal micrognathia and clubfoot was identified during the third trimester, initially raising concerns for a fetal chromosomal abnormality or hereditary syndrome. Prenatal ultrasonography did not detect other structural anomalies, and fetal movements and the gastric bubble were preserved. Although a fetal hereditary condition was considered, CDM1 was not suspected prenatally. The definitive diagnosis was made only after the infant was born with severe hypotonia and respiratory failure.

CDM1 is characterized by severe neonatal hypotonia, respiratory failure, and feeding difficulties, and is almost exclusively maternally inherited [4]. Polyhydramnios is a well-recognized prenatal finding in affected pregnancies, thought to result from impaired fetal swallowing and reduced intrauterine movement [6]. Craniofacial anomalies, such as micrognathia and orthopedic anomalies, including clubfoot, have also been reported in association with CDM1 [7,8]. However, these prenatal findings are nonspecific and commonly observed in fetal hereditary syndromes, making the diagnostic focus prone to shift toward the fetus and making it difficult to consider the possibility of an undiagnosed maternal DM1. Previous studies have reported that a substantial proportion of mothers of infants with CDM1 were either mildly symptomatic or entirely unaware of their condition at the time of delivery [4,6]. Our case is consistent with these observations and further underscores that even when prenatal findings raise suspicion for a genetic syndrome, the diagnostic workup may remain focused on the fetus while the maternal contribution is overlooked.

An important aspect of this case was the role of the postnatal maternal interview. The severity of neonatal hypotonia prompted a reassessment of the mother, which revealed symptoms of grip myotonia that had not previously been recognized as pathological. Although the mother herself had not yet undergone genetic testing at the time of this report, the combination of maternal symptoms and the infant’s genetic diagnosis strongly suggested maternal involvement. This clinical course highlights that maternal neuromuscular disorders may remain undiagnosed until they are unmasked by severe neonatal manifestations.

Furthermore, it is noteworthy that hypogammaglobulinemia was observed in both the mother and the first child in this case. The differential diagnosis for maternal hypogammaglobulinemia includes common variable immunodeficiency, other primary immunodeficiencies, and secondary causes such as nephrotic syndrome or protein-losing enteropathy. In this case, the mother underwent evaluation by internal medicine during the first pregnancy, including workup for these conditions, but no definitive diagnosis was reached. Multisystem involvement, including immune abnormalities, has been reported in DM1, and hypogammaglobulinemia in particular has been a previously recognized manifestation, observed in approximately 40-50% of cases [5,9,10]. Notably, only IgG was selectively decreased in the mother, with no other immunoglobulin abnormalities identified. This pattern is consistent with DM1, in which accelerated catabolism of IgG, but not other immunoglobulin classes, has been reported as a characteristic feature [9]. Taken together with the grip myotonia confirmed on physical examination and the infant's confirmed CDM1 diagnosis, DM1 is the most consistent explanation for the maternal hypogammaglobulinemia. Neonatal IgG levels are primarily determined by transplacental transfer of maternal IgG, and thus the low IgG levels observed in both the first and second children likely reflect the maternal hypogammaglobulinemia rather than an intrinsic immune defect in the neonates themselves. This distinction is clinically relevant, as it suggests that neonatal hypogammaglobulinemia in this context may serve as a surrogate marker of maternal DM1 rather than an independent neonatal pathology. In retrospect, the first child also exhibited several features that may have been early manifestations of DM1, including mild hypotonia at birth, low Apgar scores, slow feeding requiring prolonged hospitalization, and hypogammaglobulinemia. Although these findings were not recognized as suggestive of a neuromuscular disorder at the time, they represent potential missed diagnostic clues that, if identified earlier, might have prompted investigation of a maternal neuromuscular condition before the second pregnancy.

From a perinatal management perspective, recognition of maternal neuromuscular disorders carries important implications. Anticipating neonatal respiratory depression enables the development of delivery plans, preparation for immediate neonatal intensive care, and coordination of a multidisciplinary team comprising obstetricians, neonatologists, and genetics specialists. Furthermore, early identification allows for appropriate genetic counseling regarding recurrence risk and future pregnancies.

This case has several limitations. Most importantly, the mother had not yet undergone genetic testing at the time of this report; therefore, the diagnosis of maternal DM1 remains presumptive, based on the clinical finding of grip myotonia and the infant’s confirmed genetic diagnosis. Although genetic testing has been recommended, the family has not yet decided to proceed. Should the maternal CTG expansion be confirmed, it would strengthen the link between maternal DM1 and the clinical findings observed across both pregnancies. Conversely, a negative result would necessitate reconsideration of the etiology of the maternal grip myotonia and hypogammaglobulinemia, and the diagnosis of CDM1 in the infant would need to be attributed to a de novo expansion or paternal transmission. Second, although grip myotonia was confirmed on physical examination, other maternal symptoms may have been underreported or unrecognized, as the maternal history was obtained retrospectively after the infant's diagnosis. Additionally, as a single case report, the generalizability of our findings is inherently limited. Nevertheless, this case emphasizes that when polyhydramnios is accompanied by fetal anomalies such as micrognathia and clubfoot, the differential diagnosis should be broadened beyond fetal conditions alone. Even in the absence of a relevant medical history, a thorough maternal history may provide important clues to an underlying maternal neuromuscular disorder and contribute to more accurate prenatal counseling and improved perinatal outcomes.

## Conclusions

This case illustrates that polyhydramnios accompanied by fetal anomalies, such as micrognathia and clubfoot, may not only indicate a fetal hereditary syndrome but may also serve as a prenatal manifestation of undiagnosed maternal DM1. Nonspecific clinical features, including maternal and neonatal hypogammaglobulinemia, and subtle neonatal findings in prior pregnancies, such as mild hypotonia, low Apgar scores, and feeding difficulties, may represent overlooked diagnostic clues to an underlying maternal neuromuscular disorder. Clinicians should consider a thorough maternal history and physical examination, including assessment for myotonia, when evaluating polyhydramnios with unexplained fetal anomalies, even in the absence of a known family history of neuromuscular disease.

Early recognition of maternal DM1 enables anticipation of neonatal complications, appropriate delivery planning with multidisciplinary team coordination, and timely genetic counseling regarding recurrence risk and future pregnancies. Heightened awareness of the maternal contribution to these prenatal findings has the potential to improve both prenatal counseling accuracy and perinatal outcomes.
